# Psychiatric Comorbidity in Children and Adults with Gluten-Related Disorders: A Narrative Review

**DOI:** 10.3390/nu10070875

**Published:** 2018-07-06

**Authors:** Mahmoud Slim, Fernando Rico-Villademoros, Elena P. Calandre

**Affiliations:** 1 Division of Neurology, The Hospital for Sick Children, The Peter Gilgan Centre for Research and Learning, 686 Bay St., Toronto, ON M5G 0A4, Canada; mahmoud.slim@gmail.com; 2 Instituto de Neurociencias, Universidad de Granada, Avenida del Conocimiento s/n, 18100 Armilla, Granada, Spain; fernando.ricovillademoros@gmail.com

**Keywords:** celiac disease, non-celiac gluten sensitivity, psychiatric disorders, depression, anxiety disorders, eating disorders, ADHD, autism, psychosis

## Abstract

Gluten-related disorders are characterized by both intestinal and extraintestinal manifestations. Previous studies have suggested an association between gluten-related disorder and psychiatric comorbidities. The objective of our current review is to provide a comprehensive review of this association in children and adults. A systematic literature search using MEDLINE, Embase and PsycINFO from inception to 2018 using terms of ‘celiac disease’ or ‘gluten-sensitivity-related disorders’ combined with terms of ‘mental disorders’ was conducted. A total of 47 articles were included in our review, of which 28 studies were conducted in adults, 11 studies in children and eight studies included both children and adults. The majority of studies were conducted in celiac disease, two studies in non-celiac gluten sensitivity and none in wheat allergy. Enough evidence is currently available supporting the association of celiac disease with depression and, to a lesser extent, with eating disorders. Further investigation is warranted to evaluate the association suggested with other psychiatric disorders. In conclusion, routine surveillance of potential psychiatric manifestations in children and adults with gluten-related disorders should be carried out by the attending physician.

## 1. Introduction

Gluten-related disorders include three pathologies caused by the ingestion of gluten-containing cereals grains, namely celiac disease (CD), non-celiac gluten sensitivity (NCGS) and wheat allergy (WA) [1]. Although all of them are due to the toxicity of gluten proteins in the sensitive subject, their respective pathogenetic mechanisms differ.

Celiac disease is a systemic autoimmune disease due to a permanent intolerance to gluten which causes villous atrophy of the intestinal mucosa. It involves both innate and adaptive immune responses that appear in genetically predisposed subjects exposed to gluten and, unlike food allergies, it is not mediated by an immediate hypersensitivity reaction. It is a polygenic multifactorial disorder whose development depends on the genetic constitution of the subject, on his/her exposure to gluten intake, and on different environmental factors [2,3]. To date, the only effective treatment for the disease is to observe a life-long strict gluten-free diet although other therapeutic approaches are being explored [4].

In relation to the genetic background of the disease, two HLA class II genes, the HLA-DQ2 and the HLA-DQ8 heterodimers are present in almost all CD patients and their simultaneous absence in a subject usually rules out a diagnosis of CD. However, these genes are also common in the general population and the implication of other non-HLA genes is being investigated by genome wide association studies [5]. Environmental factors that facilitate or, conversely, protect against the development of CD are defectively known although they are considered important given that the genetic background is not enough to explain the increasing incidence and prevalence of CD [2]. Infant feeding practices such as the timing of the first gluten introduction in the diet and the presumed protective role of maternal breastfeeding that were once considered important, have been recently shown to be irrelevant in relation to the development of CD [6]. In contrast, gastrointestinal infections and antibiotics use during the first year of life seem to be associated with a higher risk of developing CD [7]; these latter factors could be related with the composition of gut microbiota that seems to be different between children with and without CD [8].

As both the two most relevant genes associated with the development of CD as well as the consumption gluten-containing foods are fairly prevalent in most of the world, it is not surprising that there is high worldwide prevalence of CD [9]. The global worldwide prevalence of CD has been shown to be higher when diagnosed only by serological tests, i.e., anti-tissue transglutaminase and/or antiendomysial antibodies (1.4%, 95% confidence interval [CI] 1.1–1.7%) than when diagnosed with intestinal biopsy (0.7%, 95% CI 0.5–0.9%) [10]. Some striking differences have been found among different geographic areas; differences that are probably due to different genetic haplotypes, different patterns of gluten-containing foods intake, and environmental differences. CD has been found to be more frequent in females than in males and in children than in adults [10]. A fact worthy of mention is that the CD prevalence has been increasing during the last decades [2,10]. This increase must be partially attributed to an augmented awareness about the disease and more accurate diagnosis, but environmental factors are also responsible for being the most relevant the increase to gluten exposure in countries where nutrition traditionally relied on the intake of gluten-free grains such as rice or corn [3]. 

The clinical manifestations of CD can be both gastrointestinal and extraintestinal. Gastrointestinal symptoms include diarrhea, steatorrhea, abdominal pain, abdominal bloating, vomiting and failure to thrive due to the malabsorption process. This kind of symptomatology is more frequent in children and was formerly called “typical CD”, a term that has currently been replaced by “classic” CD [3]. Among the extraintestinal manifestations, some of them such as ferropenic anemia, osteopenia and osteoporosis, short stature or dental enamel hypoplasia, are a consequence of the intestinal malabsorption process. Others, however, seem to be due to the noxious effect of gluten in the affected organs; dermatitis herpetiformis, gluten ataxia, gluten encephalopathy, epileptic seizures or elevation of liver enzymes are examples of the latter. Extraintestinal symptoms, which are more frequently found among adolescents and adults, were initially known as “atypical” CD, a term that has now been replaced by “symptomatic” CD [3]. 

CD is frequently comorbid with mainly other autoimmune disorders, although non-exclusively, type1 diabetes, Graves’ disease and inflammatory bowel diseases [11,12]. It has also been found to be associated with a higher risk of non-Hodgkin lymphoma [13,14] and with Down [15,16] and Turner syndromes [15,17]. 

Unlike CD, NCGS has not been shown to be associated with underlying autoimmune mechanisms. Similar to patients with CD, subjects that experience NCGS may, after gluten intake, suffer a wide variety of intestinal and/or extraintestinal symptoms that improve after following a gluten-free diet. Contrarily to CD, the presence of anti-tissue transglutaminase and/or antiendomysial antibodies is always negative, the HLA-DQ2/HLA-DQ8 combination in these patients is only slightly more frequent than in the general population, and there is no atrophy of the small intestine mucosa although a rise in intraepithelial intestinal lymphocytes has been observed [18]. Its prevalence is not yet well-known although it does not seem to be an uncommon disease [19]. The pathogenetic mechanisms of NCGS are, at present, poorly understood. Patients with NCGS benefit from a gluten-free diet but they have been also shown to improve following a low FODMAPs (fermentable, oligo-, di-, monosaccharides and polyols) diet, a fact that suggests that other constituents of grains may be responsible for the symptoms of the disease [20].

Wheat allergy is an IgE-mediated reaction to the proteins contained in wheat and in particular, although not exclusively, the omega-5-gliadin. WA can be developed by inhalation of wheat flour, the so-called baker’s asthma and baker’s rhinitis which are considered occupational diseases, or by wheat ingestion [21]. The latter case, which is the most frequent, may cause urticaria, angioedema and/or gastrointestinal symptoms such as nausea, vomiting, abdominal bloating, abdominal pain and diarrhea; in the most severe cases it can induce systemic anaphylaxis [18]. WA is especially frequent in children, being less commonly seen in adolescents and adults. The treatment is based on the avoidance of wheat-containing foods, being less restrictive compared to gluten-free diet in CD, as it does not require the restriction of rye and barley-containing foods [22]. 

Psychiatric disturbances have frequently been reported in patients with CD. Several narrative reviews of the literature undertaken in the last five years indicate that CD could be associated with a wide spectrum of psychiatric disorders, including anxiety disorders, dysthymia, major depression, bipolar disorders, schizophrenia, eating disorders, autism spectrum disorders, and attention-deficit hyperactive disorders [23,24,25,26,27]. However, these otherwise important reviews have several limitations. Several of them were focused on specific psychiatric disorders such as anxiety and depression [24], mood disorders and schizophrenia [25], or severe psychiatric disorders [27]. Some others, according to their objectives comprised the whole spectrum of psychiatric disorders, but they do not specify their search strategies and/or the biomedical literature database used for the review [23,26]. Finally, when specified, literature searches were almost restricted to PubMed, thus providing a limited review of the literature on this topic. Moreover, none of the previous have evaluated the association of psychiatric disorders in children and adults with gluten-related disorders separately. The aim of this manuscript is presenting a comprehensive review of the literature on the potential association of gluten-related disorders with the whole spectrum of psychiatric disorders using the most common literature databases for this kind of evaluation (namely, Medline, EMBASE and PyscINFO). 

## 2. Methodology

### 2.1. Search Strategy

We searched the medical literature for published studies indexed in the Medline (1966 to January 2018), EMBASE (1947 to January 2018), and PsycINFO (1967 to January 2018). The search strategy included terms of ‘celiac disease’ or ‘gluten-sensitivity related disorders’ combined with terms of ‘mental disorders’ as described in Supplementary Table S1. No limits or restrictions were applied. Retrieved references were pooled and managed using EndNote X8 (Clarivate Analytics, Philadelphia, PA, USA).

### 2.2. Inclusion Criteria

We included studies that investigated the prevalence, incidence or the likelihood of presenting mental or psychiatric disorders in patients with CD or gluten-sensitivity related disorders. For that purpose, comparative observational or interventional studies, including meta-analysis, assessing the aforementioned objectives as part of their primary or secondary objectives were included. Only studies published in English, Spanish, French, Portuguese, or Italian were included. Case-reports, case-series, abstracts and editorials were excluded. The relationship between CD and psychiatric disorders may be bidirectional. Our purpose was to assess the comorbidity between gluten-related disorders and psychiatric manifestations; thus, those studies assessing the prevalence, incidence or likelihood of presenting CD or gluten-related disorders in patients with diagnosed psychiatric disorders were excluded.

Study eligibility was independently evaluated by the three investigators (MS, EPC, FRV). Discrepancies in the evaluation were resolved by consensus among study investigators.

### 2.3. Data Extraction

Standardized data collection forms were used to extract data that included: (1) name of the first author; (2) year of publication; (3) country where the study was conducted; (4) study objective(s); (5) study design; (6) assessment tools used in psychiatric comorbidities evaluation; (7) Disease diagnostic criteria; (8) sample size and demographic characteristics; and (9) summary of outcomes. Data extraction was independently completed by two investigators (MS and FRV). Discrepancies in data extraction were solved by consensus.

## 3. Results

### 3.1. Study Selection

**Figure 1:**
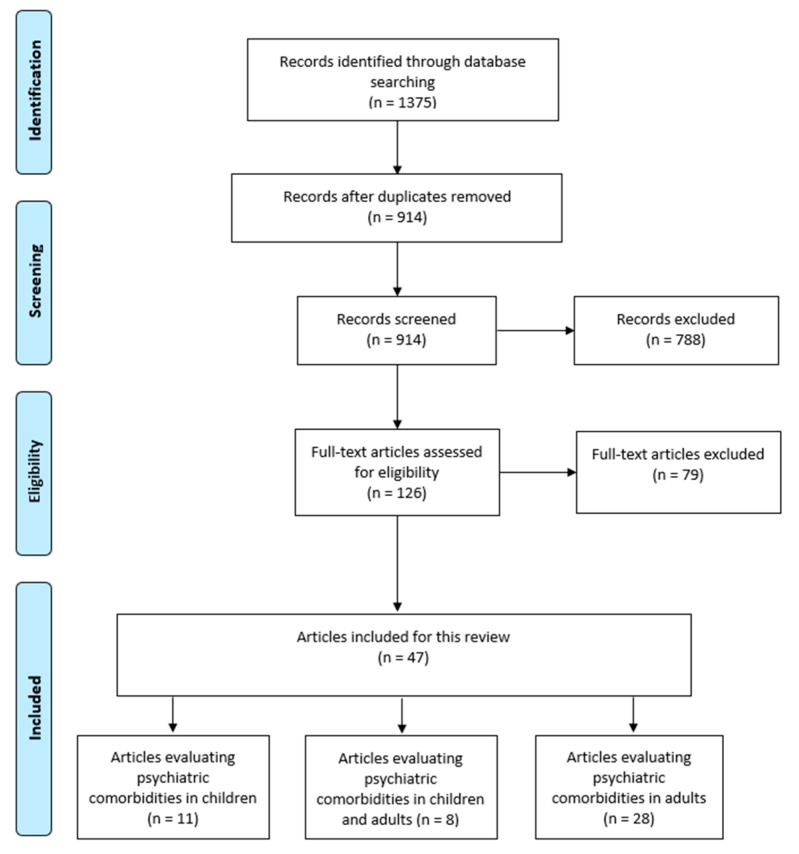
PRISMA flow chart.

Our systematic search strategy identified 1375 potentially relevant articles (730 articles from EMBASE, 453 articles from MEDLINE and 192 articles from PsycINFO). After removing 461 duplicate articles, 914 articles underwent title and abstract screening. Seven hundred and eighty-eight articles were excluded as they were case-reports, editorials, animal studies, basic science studies, did not include comparator group, or were published in a language other than those specified in the inclusion criteria, leaving 126 articles for a full-text screening. Two studies were excluded because we were unable to obtain their full text [28,29]. A total of 77 were excluded following full-text review because they were either published in abstract form, did not meet the specific objectives set for our current review or did not report outcomes of interest, leaving a total of 47 articles that were included in our review, of which 28 studies were conducted in the adult population, 11 studies were conducted in the pediatric population and eight studies included both adults and children. Mixed studies (including children and adults, *n* = 8) were classified under the corresponding population group with a larger sample size (pediatrics (*n* = 4), adults (*n* = 4)) (Figure 1). 

### 3.2. Studies Conducted in Children with CD

We found 15 studies that evaluated psychiatric disorders in children or young adults with CD, 11 of which were conducted in clinical-based settings and four were conducted in community-based settings (Table 1). Studies were published between 1997 and 2018 [30,31,32,33,34,35,36,37,38,39,40,41,42,43,44]. Most studies (*n* = 12) were cross-sectional, although one of them included a subsequent longitudinal phase [41]. Three studies used a population-based cohort design and were conducted in Sweden using the same data source for patients with CD [31,34,44]. Finally, one study used a cohort design [38]. With the exception of this later study which was conducted in several countries [38], the remaining studies were conducted in European countries or Turkey. 

According to a population-based cohort study, children with CD have a 70% increased likelihood of presenting a psychiatric disorder with intellectual disability being the most likely disorder (HR 1.7, 95% CI 1.4 to 2.1) [44]. A summary of results of studies evaluating the association between CD and the occurrence or presence of psychiatric disorders is presented in Table 2. Regarding specific conditions, cohort studies have shown that CD is associated with an increased likelihood of occurrence of depression (HR = 1.8, 95% CI 1.6 to 2.2) [34] or mood disorders (HR 1.2, 95% CI 1.0–1.4) [44], although this latter result did not reach statistical significance. In contrast, most cross-sectional studies have found that the point prevalence of depression or the severity of depressive symptoms did not differ in children with CD as compared with controls [33,35,36,37]. Pynnonen et al. [32], using a cross sectional study, found no differences between patients with CD and controls in the point prevalence of major depressive disorder, but the lifetime prevalence of major depressive disorder was significantly increased in patients with CD (31% vs. 7%; OR = 6.06, 95% CI 1.18–31.23). Although a population-based study found an increased likelihood of occurrence of anxiety disorder in patients with CD as compared with controls (HR 1.2, 95% CI 1.0 to 1.4, *p* <0.05) [44], cross-sectional studies have not shown differences between patients with CD and controls in the prevalence or severity of symptom of anxiety [32,35,36]. In children, no association has been found between CD and the occurrence of bipolar disorder [34].

The association of CD with psychotic disorders in children has been scarcely investigated, showing no association with the occurrence of schizophrenia [31] or psychotic disorder [44]; an association has been reported between CD and non-schizophrenic non-affective psychosis (HR 1.61, 95% CI 1.19–2.20) [31]. 

A population-based study found a significant association between CD and the occurrence of an eating disorder (HR 1.4, 95% CI 1.1 to 1.8) [44], and the presence of the disorder seems to have a negative impact on some dimensions of quality of life (namely, ill-being and joy-in-life) [39]. A population-based cohort found an excess likelihood of occurrence of an autism spectrum disorder in patients with CD as compared to controls [44]; however, a cross-sectional study did not find an association between both disorders [30]. A slight, but significant, increase in the likelihood of occurrence of attention deficit and hyperactive disorder (ADHD) in patients with CD has been reported [44].

Several factors have been suggested to contribute to depressive symptomatology in the pediatric population including the presence of parental depressive disorders, low parental educational level, divorce of the parents, presence of functional comorbid conditions and female gender [32,33,43]. Older age, higher body mass index and history of dietary restrictions were linked to higher risk of eating disorders [39,40].

### 3.3. Studies Conducted in Adults with CD

We found 32 studies that evaluated psychiatric comorbidities in adult patients with CD or NCGS, 18 of which were conducted in clinical-based settings and nine were conducted in community-based settings (Table 3). Studies were published between 1982 and 2018 [45,46,47,48,49,50,51,52,53,54,55,56,57,58,59,60,61,62,63,64,65,66,67,68,69,70,71,72,73,74,75,76]. More than half of these studies were of cross-sectional design [45,48,51,53,54,55,56,57,59,60,63,67,68,71,72,74,75,76] and four of them were representative of the general population [47,70,73,76].

A summary of results of studies evaluating the association between CD and the occurrence or presence of psychiatric disorders is presented in Table 4.

The prevalence rates of depression or depressive symptomatology were significantly higher in patients with CD compared to controls in the majority of the published studies except for two [56,60]. Nevertheless, significant variability in the point-prevalence of depression or depressive symptomatology exists, ranging from 14% to 68.7% [53,56,57,59,60,63]. In a meta-analysis conducted by Smith et al. [61], depression was shown to be more common and severe in CD than in healthy adults, but not compared to patients with other medical conditions. Comorbid illnesses, including type I diabetes mellitus or subclinical thyroid disease, and stress were associated with the presence of depressive symptomatology in CD [57,60]. Increased severity of gastrointestinal symptoms in CD was linked to worsened depressive symptoms [75] which, in turn, led to poorer QOL compared to controls [63]. Although gluten-free diet (GFD) did not lead to any improvement in depressive symptoms in two longitudinal studies [52,55], a meta-analysis conducted by Sainsbury et al. [66] found a moderate association between poor adherence to GFD and greater depressive symptoms. With respect to post-partum depression, it was assessed in a single study in which it turned out to be significantly more prevalent in women with CD compared to controls (41% vs. 11%, *p* < 0.01) [65].

In two studies conducted by the same research group, the prevalence of state anxiety in patients with CD was substantially higher than in controls (62.5% vs. 31.3%, and 71.4% vs. 23.7%), although the difference was statistically significant in only one study [53,55]. Generalized anxiety disorder diagnosis in CD was not shown to be prevalent in CD compared to controls [57] and the overall prevalence of anxiety was not significantly higher compared to healthy adults in the meta-analysis conducted by Smith et al. [61]. The prevalence of social phobia in CD reached 70% in one cross-sectional study [59]; however, its lifetime prevalence in another study was only 8.3% [57]. Bipolar disorder and panic disorders were significantly more prevalent in patients with CD [57,58,63].

Three studies assessed the prevalence and risk of eating disorders in CD. Their prevalence was significantly higher in adults with CD compared to healthy controls as demonstrated via the elevated scores on the different assessment scales employed in two cross-sectional studies [67,68]. Elevated Eating Attitudes Test scores were seen in around 16% of patients with CD in both studies, whereas elevated Binge Eating Scale scores were only elevated in one study with 19.7% of adults with CD reporting high scores [68]. Moreover, severe gastrointestinal symptoms were linked to greater risk of eating disorders [75]. In the register-based cohort and case-control study conducted by Marild et al. [69], the likelihood of developing anorexia nervosa was significantly higher in women with CD (HR = 1.46, 95% CI: 1.08–1.98) and the likelihood was highest in women with normal mucosa and positive serology (HR = 2.45, 95% CI: 1.1–5.45).

While patients with CD were less likely to experience alcohol-related disorders [72], their risk of developing dementia was significantly higher as shown in a population-based cohort study [70,73]. The likelihood of developing poor sleep in CD based on the use of hypnotics was significantly elevated compared to controls HR = 1.36, 95% CI: 1.3–1.41) in the population-based case-control study conducted by Marild et al. [70]. On the other hand, sleep difficulty as measured with the Patient Health Questionnaire did not differ significantly between adults with CD and controls (37.3% vs. 27.4%, *p* = 0.15) in a population-based cross-sectional study [76].

While gender did not seem to affect the prevalence rates of ADHD in CD patients [45], males with CD were less likely to experience poor sleep problems [70] or subsequent anorexia nervosa [69] and they tended to score higher on the different Psychological General Well-being Index domains [71]. Conflicting results concerning the effect of the CD onset time on psychological symptomatology were obtained; on one hand, earlier onset of CD symptoms was linked to higher prevalence of major depressive disorder in one study [57] and on the other hand, depressive symptomatology scores did not differentiate between childhood or adulthood diagnosis of CD [54]. Finally, severe gastrointestinal symptomatology significantly correlated with increased psychological manifestations [68,75].

The risk of schizophrenia in patients with CD was assessed in three studies [47,48,49]. While one population-based case-control study showed no increased risk of schizophrenia in CD (OR = 0.75, 95% CI: 0.4–1.4) [47], its risk was shown to be significantly elevated in a population-based cohort study (Incidence rate ratio = 2.11, 95% CI: 1.1–3.6) and a cross-sectional study (adjusted incidence rate = 3.6, 95% CI: 1.2–10.6) [48,49]. In a meta-analysis including four studies, an increased risk of schizophrenia among patients with CD was found (OR = 2.03, 95% CI: 1.45–2.86) [50]. With respect to autistic spectrum disorders, its risk in a population-based cohort of CD appeared to be increased with the highest risk being present in patients with normal mucosa and positive serologic findings (HR = 3.09, 95% CI: 1.99–4.8) [46]. 

ADHD was assessed in one cross-sectional study that reported an increased prevalence of this disorder in adults with CD compared to controls (20.7% vs. 10.5%, *p* <0.01) [45]. The overall psychological status in adults with CD was evaluated in one study whereby no difference in the total Psychological General Well-Being Index was found between CD and controls [71].

In the two studies that evaluated the effects of gluten ingestion in adults with NCGS [62,64], significant worsening of depressive symptomatology [64] and increase in the depression subscale scores of Spielberger State Trait Personality Inventory [62] were reported.

## 4. Discussion

Our current review of the literature revealed the existence of an association between CD and other gluten-related disorders with psychiatric disorders across different age groups. CD is primarily an autoimmune disorder that is characterized by villous atrophy of the intestinal mucosa along with intraepithelial lymphocytosis and crypt hyperplasia [77]. Nevertheless, a major shift in clinical presentation with extraintestinal manifestations becoming more prevalent than classical gastrointestinal symptoms has been suggested [78]. The reviewed data demonstrate that a wide range of psychiatric disorders have been investigated in CD and NCGS including autism spectrum disorders, schizophrenia, attention-deficit disorder, depression and mood disorders, anxiety disorders, bipolar disorder, feeding and eating disorders, sleep disorders, substance-related and addictive disorders and neurocognitive disorders.

Most of the cross-sectional studies in the pediatric population did not find any significant differences in the point prevalence of depression or anxiety disorders [32,33,35,36,37], however, these studies had several methodological limitations which mainly included small sample size (ranging between 29 and 42 children with CD), the lack of specialized psychiatric clinical assessment, and the absence of adequate blinding measures to limit assessment bias. On the other hand, two population-based cohort studies including >9000 children each provided evidence for an increased likelihood of occurrence of depression and anxiety disorders in patients with CD [34,44]. In the cohort study conducted by Ludvigsson et al. [34], it was shown that adults and children with CD are at increased risk of being diagnosed with depression but not bipolar disorder later in life (i.e., during adulthood for children diagnosed with CD), whereas in the study conducted by Butwicka et al. [44], CD was identified as a risk factor for mood disorders, anxiety disorders, eating disorders, behavioral disorders, ADHD, ASD, and intellectual disability diagnosed prior to 18 years of age. Although the analyses in the two previous cohort studies were controlled for children’s age, stratified analyses to identify the likelihood of occurrence of specific psychiatric disorders across the different age groups are worth evaluation taking into consideration the variation in clinical presentation across the developmental span between 0 and 15 years of age [79]. 

In adults, the point-prevalence of depression was significantly higher in patients with CD in the majority of published studies. These findings were ascertained by a population-based cohort study in which the HR of depression (in participants ≥16 years at diagnosis) was two folds higher than controls [34] and by a meta-analysis in which depression was shown to be more common and severe in CD than in healthy adults with an overall effect size of 0.97 [61]. A comprehensive review, evaluating the comorbidity of depression and anxiety in CD, concluded that these disorders are common disorders among patients with CD and contribute to a poorer quality of life [24]. Nevertheless, the lack of differences in the prevalence of depression when compared to patients with other physical disorders [61] raises a question about the existence of a specific underlying pathophysiological mechanism in patients with CD or whether depression represents a non-specific disorder affected by physical and psychosocial distress. The association between chronic medical diseases and depression is well-known and many different causes, including both genetic predisposition and environmental factors have been shown to be involved [80,81,82]. This association is frequently bidirectional, as the presence of physical illness often worsens the affective disorder and vice versa [81]. The current information relative to depression in patients with CD does not allow, at the present time, to ascertain the exact relationship and the predisposing factors involved between CD or NCGS and depressive symptomatology. 

The association between CD and eating disorders has been investigated in a limited number of studies. Current findings reveal an elevated prevalence of eating disorders in CD among both children and adults with CD [39,40,44,67,68,69]. These disorders encompassed anorexia nervosa, bulimia nervosa and binge eating. Poor dietary management can occur as a result of physical dissatisfaction, which is not uncommon in patients with CD [83]. Moreover, evidence from the current literature suggests that young adults with chronic illnesses that require dietary modification are at higher risk of developing pathological eating practices [39]. The elevated lifetime comorbidity between depression and eating disorders [84] could be another explanatory mechanism of increased prevalence of eating disorders in CD patients who are more prone to developing depressive symptomatology.

Concerning psychotic disorders, the current evidence provided by solely two population-based cohort studies does not support the presence of an association between these disorders and CD in children [31,44]. However, children and young adults (≥16 years of age) with CD were 1.8-fold more likely to experience non-schizophrenic non-affective psychosis [31]. The authors of the latter cohort study yet did not rule out the presence of a potential association between CD and schizophrenia as the risk of the latter disorder was high despite the low number of individuals with schizophrenia. These findings were similar to another population-based case-control study conducted in adults in which no evidence of an increased risk of schizophrenia in CD was found [47]. In contrast, Benros et al. [49] demonstrated an increased incidence of schizophrenia in patients with prior CD in their population-based cohort study. Furthermore, Eaton et al. [48] showed also 3.8-fold increase in incidence rates of prior CD diagnosis in subjects with schizophrenia. However, in the latter study, data on parents’ celiac status were also included in their analysis which might have led to biased findings. A meta-analysis including four studies demonstrated the presence of an increased risk of schizophrenia among patients with CD [50]. We believe that the pooled-effect estimate in the previous meta-analysis could be biased because their pooled analysis on one hand missed the negative findings reported by West et al. [47] and on the other hand included the findings of a study in which the prevalence of CD in patients with schizophrenia was investigated [85]. The objectives and outcome measures of the latter study [85] did not match the principal objective of the meta-analysis whereby the authors investigated the prevalence of autoimmune diseases (including CD) in patients with schizophrenia and not the other way around [85]. The association between CD and gluten-related disorders with schizophrenia has been under investigation for more than five decades but most studies evaluated the prevalence or risk of gluten-related disorders in patients already diagnosed with schizophrenia [86]. Current evidence suggests a two-fold increase in the prevalence of CD in schizophrenia patients [87] and an association between gluten ingestion and exacerbation of schizophrenia symptoms [88]; nonetheless, these findings are highly inconsistent across different clinical, immunological, and epidemiological studies [86] and have not been replicated in patients presenting with CD. 

The underlying mechanisms behind the association between CD and psychiatric disorders are not well-known. Nevertheless, several potential biological and psychosocial explanations have been suggested: (i) Several psychiatric disorders such as depression, anxiety, and ADHD, among others have been linked to certain nutritional and vitamin deficiencies [89] and it is well-known that patients with CD often suffer from malnutrition prior to diagnosis or as a result of dietary non-compliance [90]; (ii) The immune-mediated processes underlying CD have been postulated as potential causative factors of the different psychiatric manifestations taking into consideration the involvement of chronic immune system activation in the etiology of various psychiatric conditions [91]; (iii) The bidirectional communication between the gastrointestinal tract [92] and the brain may suggest that alterations in the intestinal permeability, which is cardinal manifestation in CD [93], could be eventually involved in the pathophysiology of psychiatric manifestations in patients with CD; (iv) Finally, psychosocial aspects commonly seen in CD could place this population at an increased risk of developing psychiatric disorders, for instance, the introduction of GFD is associated with radical changes in daily life activities, eating habits and lifestyle which could be particularly stressful and difficult to accept [43,94]. In addition, effective adherence to GFD entails greater burden manifested via increased daily expenditure on more expensive products, social isolation and constant fear about dietary mistakes [95].

The studies included in this review provided limited data on potential factors associated with psychiatric comorbidity in patients with CD. Bearing in mind this limitation, none of the demographic factors has been consistently associated with the presence or occurrence of psychiatric comorbidities and the role of ethnicity in this context has not been studied. Regarding clinical factors, only severity of CD symptoms appears to be associated with the presence and/or severity of psychiatric disorders [33,51,52,68,75]. In this regard, the significant positive association between increased severity of gastrointestinal symptoms and worsening of psychiatric manifestations [75] and QOL [63] in CD indirectly demonstrates the importance of adherence to GFD. Nevertheless, few studies have documented the beneficial effects of GFD on psychiatric manifestations in patients with CD [27,66], with the majority of these studies suffering from several methodological flaws limiting our capacity of reaching definitive conclusions supporting the role of GFD in this context. 

Only two studies in patients with NCGS supported the association between this relatively new entity and depressive symptomology [62,64]. It has not been until recently that standardized diagnostic criteria for NCGS were established [19], which might explain the limited number of studies investigating psychiatric comorbidities in NCGS. In our current review search, we could not find any study that investigated psychiatric comorbidities in patients with WA.

Limitations of our current review are essentially derived from the limited quality of the majority of the studies that have investigated psychiatric disorders in CD. Most of these studies are of cross-sectional design which does not allow establishing causal relationships and are of small sample size, whereas very few population-based studies have been published. 

Evaluating psychiatric comorbidities in different age groups adds strength to our current review since up to the current date, none of the previous reviews had evaluated the evidence of psychiatric disorders in children and adults with CD separately. Interestingly, according to our review, the presence of CD in childhood seems to be associated with an increased risk of developing psychiatric disorders later during adulthood, but not with an increased prevalence of these disorders during childhood.

## 5. Conclusions

Our current comprehensive review ascertains the presence of an association between CD and psychiatric disorders with varying grades of evidence from one condition to another. In our view, there is enough evidence supporting an association of CD with depression and, to a lesser extent, with eating disorders. Some studies also point out to an association between CD and panic disorder, autism and ADHD, but the evidence is limited, and these potential associations should be further investigated. Finally, the data regarding the association of CD with schizophrenia or other anxiety disorders is conflicting. Overall, psychiatric symptomatology which could be perceived as part of the atypical manifestations of this chronic condition are linked to significant distortion in quality of life and moderately increased risk of suicide [96] and thus warrants further attention. Therefore, gastroenterologists and other healthcare professionals involved in the management of patients with CD should be aware of the increased risk of psychiatric disorders in these patients. Thus, routine surveillance of potential psychiatric manifestations, especially anxiety and/or depressive symptomatology that seem to be the most common forms of disturbances, should be carried out by the attending physician in order to refer the patient to the mental health services if necessary.

## Figures and Tables

**Table 1:** Objectives and design of studies evaluating the association between gluten-related disorders and psychiatric disorders in children and young adults.

Author (Year)	Country	Primary Objective	Design ^‡^	Study Setting	Psychiatric Comorbidity Assessment	Celiac Disease Diagnostic Criteria
Autism spectrum disorders						
Pavone (1997) [30]	Italy	To evaluate behavioral problems and autistic features in children with CD	Cross-sectional	Clinical	DSM-III-R	Biopsy
Schizophrenia Spectrum						
Ludvigsson (2007) * [31]	Sweden	To determine the risk of non-affective psychosis in patients with CD in a national general population cohort	Population-based cohort	Community	ICD	ICD
Bipolar, depressive and anxiety disorders						
Pynnonen (2004) [32]	Finland	To compare the prevalence of current and lifetime mental disorders in adolescents with CD and controls	Cross-sectional	Clinical	K-SADS-PL Youth Self-Report BDI and BAI HDRS and HARS	Biopsy
Accomando (2005) * [33]	Italy	To investigate the relationship between CD and depression	Cross-sectional	Clinical	CDQ (adults) CDS (children)	NR
Ludvigsson (2007) * [34]	Sweden	To investigate the risk of subsequent depression and bipolar in patients with CD	Population-based cohort	Community	ICD	NR
Fidan (2013) [35]	Turkey	To investigate the depression and anxiety levels of children and adolescents with celiac disease and the impact of these on quality of life	Cross-sectional	Clinical	CDI STAIC	NR
Esenyel (2014) [36]	Turkey	To explore the diet compliance and depression and anxiety levels of pediatric celiac children and their families after a GFD	Cross-sectional	Clinical	CDI SCARED	ESPGHAN criteria
Simsek (2015) [37]	Turkey	To evaluate depressive symptoms at time of CD diagnosis and 6 months following GFD initiation	Phase 1: Cross-sectional Phase 2: Case-series	Clinical	CDI HRQOL (Kid-KINDL)	Biopsy
Smith (2017) [38]	USA, Finland, Germany, and Sweden	To assess mother’s report of psychological functioning in children with CDA	Cohort	Community	CBCL	Serology and optional biopsy
Feeding and eating disorders						
Wagner (2015) [39]	Austria	To assess the determinants of eating disorders in female adolescents with CD	Cross-sectional	Clinical	EDI-2 EDE DSM-IV for subclinical eating disorders CDI (total score ≥ 18)	Both
Babio (2018) * [40]	Spain	To assess the risk of eating disorders in individuals between 10 and 23 years old diagnosed with CD	Cross-sectional	Clinical	CEAT EAT-26 SCFF BITE BSQ	Both
Overall psychological status						
Terrone (2013) [41]	Italy	To screen for neurological and behavioral disorders in children with CD	Phase 1: cross-sectional Phase 2: cohort	Clinical	PSC (total score ≥ 28)	ESPGHAN criteria
Various psychiatric conditions						
Ruggieri (2008) [42]	Italy	To determine the prevalence of neurologic symptoms in children with gluten sensitivity enteropathy	Cross-sectional	Clinical	NR	Both
Mazzone (2011) [43]	Italy	To identify psychological features in children with CD following strict GFD	Cross-sectional	Clinical	MASC CBCL CDI DSM-IV-TR criteria to assess autistic disorders	ESPGHAN criteria
Butwicka (2017) [44]	Sweden	To examine the risk of psychiatric disorders in children with a biopsy-verified diagnosis of CD and to examine the prevalence of psychiatric disorders before CD is diagnosed in children	Population-based cohort	Community	ICD	Biopsy

* Included patients of all age groups (pediatrics and adults); **^‡^** The design was determined by the authors of the current review which might not coincide with the design described in the original studies; for studies including multiple methodologies, the design that achieved the objectives of interest was selected; BAI: Beck Anxiety Inventory; BDI: Beck Depression Inventory; BITE: Bulimia Investigatory Test Edinburgh; BSQ: Body Shape Questionnaire; CBCL: Achenbach Child Behavior Checklist; CD: celiac disease; CDA: celiac disease autoimmunity; CDI: Child Depression Inventory; CDQ: Clinical Depression Questionnaire; CDS: Children Depression Scale; CEAT: Children Eating Attitudes Test; DSM: Diagnostic and Statistical Manual of Mental Disorders; EAT: Eating Attitudes Test; EDE: Eating Disorder Examination; EDI: Eating Disorder Inventory; ESPGHAN: The European Society for Pediatric Gastroenterology, Hepatology, and Nutrition; GFD: gluten-free diet; HARS: Hamilton Anxiety Rating Scale; HDRS: Hamilton Depression Rating Scale; HRQOL: Health-Related Quality of Life; ICD: International Classification of Disease; KINDL: German questionnaire for measuring quality of life in children and adolescents; K-SADS-PL: Schedule for Affective Disorders and Schizophrenia for school-Age Children-Present and Lifetime version; MASC: Multidimensional Anxiety Scale for Children; NR: not reported; PSC: Pediatric Symptom Checklist; SCARED: Childhood Anxiety Disorders Screening Measure; SCFF: Sick Control Fat Food; STAIC: State-Trait Anxiety Inventory for Children.

**Table 2:** Summary of outcomes evaluating the association between gluten-related disorders and psychiatric disorders in children and young adults.

Author (Year)	Design	Sample Size and Demographic Characteristics	Summary of Outcomes	Associated Factors with Psychiatric Comorbidities and Other Relevant Information
Autism spectrum disorders				
Pavone (1997) [30]	Cross-sectional	CD, *n* = 120 (mean age 9.6 years, 48% females) Recently-diagnosed CD, *n* = 27 CD on strict GFD, *n* = 70 GFD non-adherent CD, *n* = 23 Controls, *n* = 20 (mean age 9.6 years, 48% females)	- Autism diagnosis: none of the recently-diagnosed CD - Language delay: Two subjects in GFD-compliant, one subject in the non-adherent group - Differences were not statistically significant compared to controls	NR
Schizophrenia Spectrum				
Ludvigsson (2007) [31]	Population-based cohort study	CD, *n* = 14,003 (age at diagnosis, 0–15 years 66% & ≥16 years 34%; 59% females) Controls, *n* = 68,125 (matched age and gender)	- Likelihood of psychosis in CD vs. controls using a Cox regression model stratified for gender, age, year of study entry and county: Any non-affective psychosis (schizophrenia and other psychoses) HR = 1.55 (95% CI: 1.16–2.06) Non-schizophrenic non-affective psychosis HR = 1.61 (95% CI: 1.19–2.20) Schizophrenia HR = 1.43 (95% CI: 0.77–2.67)	NR
Bipolar, depressive and anxiety disorders				
Pynnonen (2004) [32]	Cross-sectional	CD, *n* = 29 (mean age 14.2 years, 55% females) Controls, *n* = 29 (mean age 14.4 years, 55% females)	- Lifetime prevalence of major depression disorder (CD vs. controls): 31% vs. 7%, *p* <0.05. OR = 6.06 (95% CI: 1.18–31.23). - Disruptive behavior disorders (CD vs. controls): 28% vs. 3%, *p* <0.05. OR = 10.67 (95% CI: 1.24–92). - Lifetime prevalence of anxiety disorders (CD vs. controls): 21% vs. 24%, *p* = NS - Differences in the prevalence of current depressive, anxiety, or disruptive behavior disorders between the two groups were non-significant	- History of parental depressive disorder was more common in CD patients with depressive symptomatology compared to CD without depressive symptomatology - Parental educational level, divorce of parents, poor weight or height gain, and somatic symptoms were not associated with mental disorders
Accomando (2005) [33]	Cross-sectional	CD, *n* = 42 (17 adults and 25 children) HC, *n* = 42	Prevalence of depression (CD vs. HC): 26.2% vs. 30.9%, *p* = NS	- Females predominated in CD patients with depression (not reaching statistical significance) - Depression was more common in CD with functional comorbid conditions (specific conditions not specified)
Ludvigsson (2007) [34]	Population-based cohort study	CD, *n* = 13,776 (median age at diagnosis 2 years, 58.6% females) Controls, *n* = 66,815 (median age at diagnosis 2 years, 58.7% females)	- CD was associated with an increased risk of subsequent depression (HR = 1.8, 95% CI: 1.6–2.2) - No significant association between CD and bipolar disorder was reported (HR = 1.1, 95% CI: 0.7–1.7)	- Socioeconomic index didn’t have any confounding effect on the later schizophrenia diagnosis in CD
Fidan (2013) [35]	Cross-sectional	CD, *n* =30 (mean age 12.4 ± 3.1 years, 57% females). HC, *n* = 30 (mean age NR, 57% females)	- CD vs. HC: CDI: 10.8 ± 7.4 vs. 8.8 ± 6.8, p=0.28 STAIC-State Anxiety: 34.6 ± 6.1 vs. 32.8 + 7.2, *p* = 0.30 STAIC-Trait Anxiety: 33.7 ± 6.5 vs. 33 ± 6.3, *p* =0.64	- Data on the impact of depression and anxiety on HRQOL NR
Esenyel (2014) [36]	Cross-sectional	CD, *n* = 30 (mean age 11.9 ± 2 years, 70% females) HC, *n* =20 (mean age 12 ± 2 years, 55% females)	- CD vs. HC: CDI points: 8.73 ± 5.51 vs. 8.3 ± 4.02, *p* = 0.921 SCARED points: 24.5 ± 14.41 vs. 17.85 ± 9.12, *p* = 0.120 - There were no differences in depression and anxiety scores between patients with CD compliant or non-compliant with a GFD	NR
Simsek (2015) [37]	Phase 1: Cross-sectional Phase 2: Case-series	CD, *n* = 25 (mean age 11.8 years, 72% females) Controls, *n* = 25 (mean age 12.2 years, 64%)	- At the time of diagnosis (CD vs. controls): CDI scores: 9 vs. 6, *p* = NS - 6 months following GFD initiation: CDI scores in CD: 9 before diet vs. 9.5 after diet, *p* = NS	- Total scores of HRQOL were significantly lower in CD patients (*p* <0.05)
Smith (2017) [38]	Cohort	Aware-CDA, *n* = 440 (58% females) Unware-CDA, *n* = 66 (50% females) No CDA, *n* = 3651 (NR)	- At 3.5 years of age, unaware-CDA mothers reported more anxious/depressed symptoms, aggressive behavior, and externalizing composite score compared to aware-CDA group (*p* <0.05) or without CDA (*p* <0.05) - At 3.5 years of age, Aware-CDA mothers reported significantly fewer problems on the anxious/depressed subscale compared to No CDA group (*p* = 0.03) - At 4.5 years, there were no significant differences	NR
Feeding and eating disorders				
Wagner (2015) [39]	Cross-sectional	CD, *n* = 206 (mean age NR) CD with ED, *n* = 32 (mean age 16.4 yeas) CD without ED, *n* = 174 (mean age 14.5 years) Controls, *n* = 53 (mean age 14.7 years)	- Lifetime prevalence of EDs: 5.3% of girls with CD: anorexia nervosa (*n* = 1), bulimia nervosa (*n* = 4), and EDs not otherwise specified (*n* = 6); 3.9% suffered from current ED - Criteria for lifetime subclinical EDs: 21 girls (10.2%) with CD - Higher BMI and self-directedness were predictors of greater risk of ED - Higher ill-being and lower joy in life were reported by patients with CD with ED compared with patients without EDs, even when controlling for age and depression levels	- No differences between patients (with CD) with and without EDs in coping strategies were found - Higher BMI and lower self-directedness were linked to higher risk of ED in CD
Babio (2018) [40]	Cross-sectional	CD, *n* = 98 (mean age 15 years, 60% females) Controls, *n* = 98 (mean age 15 years, 60% females)	- No significant differences in the median scores of the screening tools for EDs between CD and HC - CD vs. HC: β coefficient = 2.15 (1.04); *p* = 0.04 in a multiple linear regression model for EAT after adjusting for several factors	- Only significant results for one out of the 4 models (one for each screening test) - Age > 13 years old was positively associated with an increase in the score on the EAT
Overall psychological status				
Terrone (2013) [41]	Phase 1: cross-sectional Phase 2: cohort	CD, *n* = 139 (mean age 10 years, 64.7% females): Group A (*n* =40): newly diagnosed CD Group B (*n* = 54): CD in remission on GFD > 1 year Group C (*n* = 45): potential CD	- Comparison of mean PSC scores using ANOVA: Group A, 14.8 ± 4.2 (one pathological score) vs. Group B, 12.3 ± 6.4 (one pathological score) vs. Group C, 7.6 ± 6 (*p* <0.0001)	NR
Various psychiatric conditions				
Ruggieri (2008) [42]	Cross-sectional	GS, *n* = 835 (demographic characteristics NR) Controls, *n* = 300 (demographic characteristics NR)	- 3 out of 835 children had bipolar disorders - None of the controls had psychiatric disorders	NR
Mazzone (2011) [43]	Cross-sectional	CD, *n* = 100 (mean age 10.4 years, 65% females) HC, *n* = 100 (mean age 11.5 years, 58% females)	- MASC scores: CD children showed significantly higher scores (50 ± 8.3 vs. 42.9 ± 6.6, *p* <0.01) - CDI scores: CD children showed significantly higher scores (8.1 ± 5.7 vs. 5.6 ± 3.4, *p* <0.01) - No significant differences were found in CBCL analysis - Two children in the CD group were classified within the spectrum of autistic disorders	- CD males showed significantly higher scores for total CBCL - CD females showed an increased rate of anxiety and depression symptoms, as indicated by significantly higher MASC and CDI scores
Butwicka (2017) [44]	Population-based cohort study	CD, *n* = 10,903 (median age 3 years, 62% females) Controls, *n* = 1,042,072 (age NR but matched, 61% females)	- HRs from a Multivariate Cox regression adjusted for maternal/paternal age at child’s birth, maternal/paternal country of birth, level of education of higher-educated parent, gestational age, birth weight, birth cohort, Apgar score, and history of psychiatric disorders before recruitment: Any psychiatric disorder 1.4 (95% CI: 1.3–1.4) Psychotic disorders 1.9 (95% CI: 1.0–3.5) Mood disorders 1.2 (95% CI: 1.0–1.4) Anxiety disorders 1.2 (95% CI: 1.0–1.4) EDs 1.4 (95% CI: 1.1–1.8) Substance misuse 1.0 (95% CI: 0.9–1.3) Behavioral disorders 1.4 (95% CI: 1.2–1.6) ADHD 1.2 (95% CI: 1.0–1.4) Autism spectrum disorder 1.3 (95% CI: 1.1–1.7) Intellectual disability 1.7 (95% CI: 1.4–2.1)	NR

ADHD: Attention-Deficit Hyperactivity Disorder; ANOVA: analysis of variance; BMI: body mass index; CBCL: Achenbach Child Behavior Checklist; CD: celiac disease; CDA: celiac disease autoimmunity; CDI: Child Depression Inventory; CI: confidence interval; EAT: Eating Attitudes Test; ED: eating disorder; GFD: gluten free diet; GS: gluten sensitivity; HC: healthy controls; HR: hazard ratio; HRQOL: Health-Related Quality of Life; MASC: Multidimensional Anxiety Scale for Children; NR: not reported; NS: not significant; OR: odd ratio; PSC: Pediatric Symptom Checklist; SCARED: Childhood Anxiety Disorders Screening Measure; STAIC: State-Trait Anxiety Inventory for Children; vs: versus.

**Table 3:** Objectives and design of studies evaluating the association between gluten-related disorders and psychiatric disorders in adults.

Author (Year)	Country	Primary Objective	Design ^‡^	Study Setting	Psychiatric Comorbidity Assessment	Celiac Disease Diagnostic Criteria
Attention-Deficit/Hyperactivity Disorder						
Zelnik (2004) * [45]	Israel	To evaluate neurologic disorders including ADHD in CD	Cross-sectional	Clinical	DSM criteria for ADHD	Both
Autism spectrum disorders						
Ludvigsson (2013) * [46]	Sweden	To examine the association between autistic spectrum disorder and CD	Cohort study	Community	ICD	Group 1: villous atrophy, Marsh stage 3 Group 2: villous atrophy, Marsh stages 1–2 Group 3: normal mucosa and positive serologic findings
Schizophrenia Spectrum						
West (2006) [47]	UK	To compare the risk of schizophrenia in patients with CD, ulcerative colitis, Crohn’s disease with the general population	Population-based cross-sectional	Community	NR	NR
Eaton (2006) [48]	Denmark	To estimate the association of schizophrenia with autoimmune disorders	Cross-sectional	Community	ICD	ICD
Benros (2011) [49]	Denmark	To investigate whether autoimmune diseases are associated with increased risk of schizophrenia	Population-based retrospective cohort	Community	ICD	NR
Wijarnpreecha (2018) [50]	USA	To evaluate the risk of developing schizophrenia among patients with CD	Meta-analysis	NA	NR	NR
Bipolar, depressive or anxiety disorders						
Hallert (1982) [51] Hallert (1983) [52]	Sweden	To compare the prevalence of psychiatric illness among patients with CD vs. controls and to assess the effects of gluten withdrawal and vitamin B6 supplement on depressive symptoms	Phase 1: cross-sectional Phase 2: case-series	Clinical	MMPI	Both (serological and biopsy) combined with morphological improvement with GFD
Addolorato (1996) [53]	Italy	To conduct psychometric evaluation in patients with CD or IBD compared to healthy controls	Cross-sectional	Clinical	STAI IDSQ	Both
Ciacci (1998) [54]	Italy	To explore the relevance of depressive symptoms in a large series of adult celiacs	Cross-sectional	Clinical	SRDS	Both
Addolorato (2001) [55]	Italy	To evaluate state and trait anxiety and depression in adult CD patients before and after 1 year of GFD	Phase 1: Cross-sectional Phase 2: Case-series	Clinical	STAI SRDS	Both
Cicarelli (2003) [56]	Italy	To evaluate the prevalence of headache, mood disorders, epilepsy, ataxia and peripheral neuropathy in adult celiac patients	Cross-sectional	Clinical	DSM-IV	Both
Carta (2002) [57] Carta (2003) [58]	Italy	To evaluate the association between celiac disease and specific anxiety and depressive disorders	Cross-sectional	Clinical	CIDI-DSM-IV	Both
Addolorato (2008) [59]	Italy	To evaluate social phobia in CD patients	Cross-sectional	Clinical	LSAS total > 30 SRDS > 49	Both
Garud (2009) [60]	US	To determine the prevalence of psychiatric and autoimmune disorders in patients with CD in the US compared with control groups	Cross-sectional	Community	Clinical charts	Biopsy
Smith (2012) [61]	Denmark	To investigate whether CD is reliably linked with anxiety and/or depression	Meta-analysis	NA	NA	NA
Peters (2014) [62]	Australia	To investigate the effect of gluten on mental state among patients with NCGS	Randomized, double-blind, cross-over trial	Clinical	STPI	Challenging with varying amounts of gluten
Carta (2015) [63]	Italy	To measure the association between CD and affective disorders	Cross-sectional	Clinical	DSM-IV	NR
Di Sabatino (2015) [64]	Italy	To assess the effects of gluten administration on intestinal and extraintestinal symptoms in subjects with NCGS	Randomized, double-blind, placebo-controlled cross-over trial	Clinical	Extraintestinal symptoms, including depression, were self-reported by patients as absent or present	Self-reported persistence of relevant intestinal and extraintestinal symptoms at low gluten doses
Tortora (2013) [65]	Italy	To evaluate the prevalence of post-partum depression in CD	Cross-sectional	Clinical	EPDS (Total score > 10 possible PPD)	Both
Sainsbury (2018) [66]	UK	To synthesize the evidence on the relationship between depression and degree of adherence to GFD in patients with CD	Meta-analysis	NA	NA	NA
Feeding and eating disorders						
Passananti (2013) [67]	Italy	To investigate the prevalence of eating disorders in patients with celiac disease	Cross-sectional	Clinical	Structured psychological assessment using: BES (Total score ≥ 17) EAT-26 (Total score ≥ 20) EDI-2 M-SDS (Total score > 44) SCL-90	Both
Satherley (2016) [68]	United Kingdom	To examine the prevalence of eating disorders in women with CD	Cross-sectional	Clinical	EAT-26 (Total score > 20) BES (Moderate bingeing, score > 17; severe bingeing, score > 27) DASS-21	Self-reported a biopsy-confirmed diagnosis
Mårild (2017) * [69]	Sweden	To determine whether women with CD are at increased risk of diagnosis of anorexia nervosa	Register-based cohort study	Community	ICD	Group 1: villous atrophy, Marsh stage 3 Group 2: villous atrophy, Marsh stages 1–2 Group 3: normal mucosa and positive serologic findings
Sleep-Wake disorders						
Marild (2015) * [70]	Sweden	To estimate the risk of repeated use of hypnotics among individuals with CD as a proxy measure for poor sleep	Population-based cohort study	Community	Prescribed Drug Register in Sweden—Use of hypnotics	Biopsy
Substance-related and addictive disorders						
Roos (2006) [71]	Sweden	To assess psychological well-being in adults with CD with proven remission (treated for 10 years)	Cross-sectional	Clinical	PGWB	Remission was ascertained with a return of villous structure at repeat biopsy (82%) or negative serology (18%)
Gili (2013) [72]	Spain	To study the impact of alcohol disorders on length of hospital stays, over-expenditures during hospital stays, and excess mortality in CD patients	Cross-sectional	Clinical	ICD	ICD
Neurocognitive Disorders						
Lebwohl (2016) [73]	Sweden	To determine whether patients with CD have an increased risk of dementia	Population-based cohort	Community	ICD	Biopsy
Various psychiatric conditions						
Fera (2003) [74]	Italy	To estimate the incidence of psychiatric disorders in celiac disease patients on gluten withdrawal	Cross-sectional	Clinical	DSM-IV criteria	Biopsy & Clinical history
Sainsbury (2013) [75]	Australia	To compare the relevant impact of psychological symptoms to known negative impacts of gastrointestinal symptoms and adherence to the GFD on quality of life	Study 1: Cross-sectional Study 2: Cross-sectional	Clinical	DASS EDI-3 CISS	Biopsy
Zylberberg (2017) [76]	US	To assess the prevalence of depression and insomnia among patients with CD, both diagnosed and undiagnosed, and people without CD who avoid gluten	Population-based cross-sectional	Community	PHQ-9 (Total score on questions 1–9 ≥ 10) SDQ	Diagnosed CD: self-reported diagnosis Undiagnosed CD: serology

* Included patients of all age groups (pediatrics and adults); ^‡^ The design was determined by the authors of the current review which might not coincide with the design described in the original studies; for studies including multiple methodologies, the design that achieved the objectives of interest was selected; ADHD: Attention-deficit/hyperactivity disorder; BES: Binge Eating Scale; CD: celiac disease; CDS: Children Depression Scale; CIDI-DSM-IV: Composite International Diagnostic Interview for DSM-IV; CISS: Coping Inventory for Stressful Situations; DASS: Depression Anxiety Stress Scale; DSM: Diagnostic and Statistical Manual of Mental Disorders; EAT: Eating Attitudes Test; EDI: Eating Disorder Inventory; EDRS: Eating Disorder Risk Scale; EPDS: Edinburgh Postnatal Depression Scale; GFD: gluten-free diet; HADS: Hospital Anxiety and Depression Scale; IBD: inflammatory bowel disease; ICD: International Classification of Disease; IDSQ: Ipat Depression Scale Questionnaire; LSAS: Liebowitz Social Anxiety Scale; MMPI: Minnesota Multiphasic Personality Inventory; M-SDS: Modified Zung Self-Rating Depression Scale; NA: not applicable; NCGS: non-celiac gluten sensitivity; NA: not applicable; NR: not reported; PGWB: Psychological General Well-being; PHQ: Patient Health Questionnaire; PPD: post-partum depression; PSS: Perceived Stress Scale; SCL: Symptom Check List; SDQ: Sleep Disorder Questionnaire; SRDS: Zung Self-Rating Depression Scale; STAI: State and Trait Anxiety Inventory; STAI: State and Trait Anxiety; STPI: Spielberger State Trait Personality Inventory.

**Table 4:** Summary of outcomes evaluating the association between gluten-related disorders and psychiatric disorders in adults.

Author (Year)	Design	Sample Size and Demographic Characteristics	Summary of Outcomes	Associated Factors with Psychiatric Comorbidities and other Relevant Information
Attention-Deficit/Hyperactivity Disorder				
Zelnik (2004) [45]	Cross-sectional	CD, *n* = 111 (mean age 20.1 years, 57.7% females) Controls, *n* = 211 (mean age 20.1 years, 59.7% females)	- ADHD diagnosis: 20.7% in CD vs. 10.5% in controls (*p* <0.01) CD, 20.3% of female patients and 21.2% of male patients Controls, 8.7% of females and 12.9% males	-No gender differences were found in the prevalence of ADHD in patients with CD -Differences in ADHD were not different among CD patients presenting with infantile form of CD or late-onset symptoms
Autism spectrum disorders				
Ludvigsson (2013) [46]	Cohort study	Group 1, *n* = 26,995 (age at diagnosis, 0–19 years 40.4%, >20 years 59.6%; 62.1% females); Controls, *n* = 134,076 (matched age and gender) Group 2, *n* = 12,304 (age at diagnosis, 0–19 years 8.9%, >20 years 91.1%; 56.9% females); Controls, *n* = 60,654 (matched age and gender) Group 3, *n* = 3719 (age at diagnosis, 0–19 years 25.3%, >20 years 74.7%; 62.1% females; 62.1% females); Controls, *n* = 18,478 (matched age and gender)	- Risk of later ASD diagnosis: Group 1: HR = 1.39 (95% CI: 1.13–1.71) Group 2: HR =2.01 (95% CI: 1.29–3.13) Group 3: HR = 3.09 (95% CI: 1.99–4.8)	NR
Schizophrenia Spectrum				
West (2006) [47]	Population-based case-control	CD, *n* = 4732; matched controls, *n* = 23,620 Crohn’s disease, *n* = 5961; matched controls, *n* = 29,843 Ulcerative colitis, *n* = 8301; matched controls, *n* = 41,589 Demographics NR	- Prevalence of schizophrenia 0.25% in CD, 0.27% in Crohn’s disease and 0.24% in ulcerative colitis, 0.37% in general population - ORs for schizophrenia compared to controls adjusted for smoking status: 0.76 (95% CI: 0.4-1.4) in CD, 0.74 (95% CI: 0.4–1.3) in Crohn’s disease, 0.71 (95% CI 0.4–1.1) in ulcerative colitis	NR
Eaton (2006) [48]	Cross-sectional	Schizophrenia, *n* = 7704, 25 controls for each case. Demographics NR	- Prior CD diagnosis in subjects with schizophrenia: Crude incidence rate: 3.8 (95% CI: 1.3–11) Adjusted incidence rate: 3.6 (95% CI: 1.2–10.6)	NR
Benros (2011) [49]	Population-based cohort	Schizophrenia, *n* = 39,076: Prior diagnosis of autoimmune disease, *n* = 927, autoimmune disease and infections, *n* = 444, without autoimmune disease, *n* = 37,705 Demographics NR	- The risk of schizophrenia among individuals with CD was increased: CD without infection: Incidence rate ratio = 2.11 (95% CI: 1.09–3.61) CD with infections: Incidence rate ratio = 2.47 (95% CI: 1.13–4.61)	NR
Wijarnpreecha (2018) [50]	Meta-analysis	Four studies were included	- Higher risk of schizophrenia among patients with CD was found; pooled OR = 2.03 (95% CI: 1.45–2.86)	NR
Bipolar, depressive and anxiety disorders				
Hallert (1982) [51] Hallert (1983) [52]	Phase 1: cross-sectional Phase 2: case-series	CD, *n* = 12 (mean age 47 years, 67% females) Controls undergoing cholecystectomy, *n* = 12 (mean age 47 years, 67% females)	- MMPI depression subscale: Significantly higher scores in CD vs. controls (70.3 ± 12.5 vs. 59.2 ± 9.3, *p* <0.01) - MMPI sores: Post-remission in small intestinal mucosa following GFD in CD: no improvement in mood (70 ± 12.5 at point 0 vs. 68 ± 14 at year 1, *p* = NS) - Post-cholecystectomy in controls: No change in MMPI scores - Supplementation with Vitamin B6 80 mg/day for 6 months: Significant decrease in depressive symptoms (68 ± 14.0 to 56 ± 8.5, *p* <0.01)	-In patients with CD, significant correlation was found between depression scores and degree of steatorrhea -No correlation was found between abdominal complaints (diarrhea and pain) and depression scores
Addolorato (1996) [53]	Case-control	CD, *n* = 20 (mean age 37 years, 56% females) IBD, *n* = 16 (mean age 32 years, 56% females) Controls, *n* = 16 (mean age 35 years, 56% females)	- Prevalence of State anxiety: 62.5% in CD, 50% in IBD, and 31.3% in controls (*p* = NS). - Prevalence of depression: 68.7% in CD, 37.5% in IBD, and 18.8% in controls (*p* <0.01 for CD vs. controls only)	NR
Ciacci (1998) [54]	Cross-sectional	CD, *n* = 92 (mean age 29.4 years, 70% females) CPH, *n* = 48 (mean age 31.8 years, 34% females) Controls, *n* =100 (mean age 30 years, 71% females)	- Mean scores of the M-SDS: CD: 31.81 ± 7.84 CPH: 28.73 ± 7.09 (*p* = 0.038 vs. CD) Controls: 27.14 ± 5.26 (*p* <0.0001 vs. CD)	- Demographic characteristics did not influence M-SDS scores -Depressive symptoms are present to a similar extent in patients with childhood- and adulthood-diagnosed CD
Addolorato (2001) [55]	Phase 1: Cross-sectional Phase 2: Case-series	CD, *n* = 35 (mean age 29.8 years, 60% females) Controls, *n* = 59 (mean age 31.7 years, 54% females)	*Before diet:* - Prevalence of high levels of state anxiety: CD vs. control: 71.4% versus 23.7% (*p* <0.0001) - Prevalence of high levels of trait anxiety: CD vs. controls: 25.7% versus 15.2% (*p* = NS) - Prevalence of depression CD vs. controls: 57.1% versus 9.6% (*p* <0.0001) *After 1 year of GFD (T0 vs. T1)* - Prevalence of high levels of state anxiety: T0 71.4% versus T1: 25.7% (*p* <0.001) - Prevalence of high levels of trait anxiety: T0: 25.7% versus T1: 17.1% (*p* = NS) - Prevalence of depression T0: 57.1% versus T1:45.7 (*p* = NS)	NR
Cicarelli (2003) [56]	Cross-sectional	CD, *n* = 176 (mean age 30.9 years, 75% females) Controls, *n* = 52 (mean age 31.7 years, 65% females)	- Prevalence of mood disorders (CD vs. controls): Mood disorders 50 (29%) vs. 9 (17%), *p* = NS Depression episodes 24 (14%) vs. 7 (13%), *p* = NS Dysthymia 26 (15%) vs. 2 (4%), *p* <0.05	- Adherence to a strict gluten-free diet was associated with a significant reduction of dysthymia
Carta (2002) [57] Carta (2003) [58]	Cross-sectional	CD, *n* = 36 (mean age 41.1 years, 75% females) Controls, *n* = 144 (mean age 41.3 years, 75% females)	- Lifetime prevalence of psychiatric disorders (cases vs. controls): Major depressive disorder 15 (41.7%) vs. 30 (20.8%), *p* = 0.01 Dysthymic disorder 3 (8.3%) vs. 2 (1.4%), *p* = 0.05 Adjustment disorders 11 (30.5%) vs. 11 (7.6%), *p* = 0.001 Generalized anxiety disorder 10 (27.7%) vs. 23 (16%), *p* = NS Panic disorder 5 (13.9%) vs. 3 (2.1%), *p* = 0.001 Specific phobia 1 (2.7%) vs. 6 (4.2%), *p* = NS Social phobia 3 (8.3%) vs. 10 (6.9%), *p* = NS Recurrent brief depression 36.1% versus 6.9% (OR = 7.6; 95% CI: 3.2–17.8)	- Earlier onset of CD was linked to higher prevalence of major depressive disorder - Subclinical thyroid disease appears to represent a significant risk factor for these psychiatric disorders
Addolorato (2008) [59]	Cross-sectional	CD, *n* = 40 (mean age 38 years, 86% females) HC, *n* = 50 (mean age 36 years, 80% females)	- Prevalence of social phobia: 70% in CD vs. 16% in HC (*p* <0.0001) - Prevalence of depression: 53% in CD vs. 8% in HC (*p* <0.0001)	- The prevalence of social phobia or depression in patients with CD did not differ among subjects newly diagnosed with CD and those already on GFD
Garud (2009) [60]	Cross-sectional	CD, *n* = 600 (mean age 54 males & 49 females, 75% females) IBS, *n* = 200 (mean age 48 males & 45 females, 75% females) Controls, *n* = 200 (mean age 52 males & 47 females, 75% females)	Prevalence of depression: 17.2% in CD vs. 18.5% in IBS (*p* = 0.74 vs. CD) and 16% in controls (*p* = 0.79 vs. CD)	- Among CD patients, type I diabetes mellitus was identified as a significant risk factor for depression (*p* <0.01) with 37% of patients with both CD and type I DM having clinical depression
Smith (2012) [61]	Meta-analysis	Eleven studies on depression and eight studies on anxiety were included	- Depression is more common and severe in CD than in healthy adults with an overall effect size of 0.97 - Anxiety did not differ significantly between CD and healthy adults - No differences in depression or anxiety in CD vs. other medical disorders	- Other medical conditions included: Crohn’s disease, DM, IBD, lactose intolerance, surgery patients, CPH
Peters (2014) [62]	Randomized, double-blind, cross-over trial	NCGS, *n* = 22 (median age 48 years, 77% females)	- Gluten ingestion effect on STPI depression scores: Significantly higher scores in CD vs. controls (mean difference = 2.03, 95% CI: 0.55–3.51, *p* = 0.01) - No differences in other STPI state indices or for any STPI trait measures	NR
Carta (2015) [63]	Cross-sectional	CD, *n* = 46 (mean age 41 years, 83% females) Controls, *n* = 240 (mean age 41 years, 83% females)	- Prevalence of depression: 30.0% in CD vs. 8.3% in controls, *p* <0.0001 - Prevalence of panic disorder: 18.3% in CD vs. 5.4% in controls, *p* <0.001 - Prevalence of bipolar disorder: 4.3% in CD vs. 0.4% in controls, *p* <0.005	- Patients with CD but without comorbidity with major depression, panic disorder, or bipolar disorder do not show worse QOL than controls
Di Sabatino (2015) [64]	Randomized, double-blind, placebo-controlled cross-over trial	NCGS, *n* = 61 (mean age 39 years, 87% females) randomly assigned to: Gluten 4.375 mg/day for 1 week Placebo 4.375 g/day rice starch for 1 week Wash-out period: 1 week	- Depression was significantly worsened by gluten ingestion (*p* = 0.02)	NR
Tortora (2013) [65]	Cross-sectional	CD, *n* = 70 (mean age 33 years) Controls, *n* = 70 (mean age 32 years)	- EPDS scores in CD women vs. controls: 9.9 ± 5.9 vs. 6.7 ± 3.7, *p* <0.01 - EPDS > 10: 47% in CD vs. 14% in controls (OR = 3.3, *p* <0.01) - PPD diagnosis: 41% of CD women with vs. 11% in controls (*p* <0.01)	- A significant association was observed between the onset of PPD and a previous menstrual disorder in women suffering from CD - QOL scores were significantly higher in women with CD
Sainsbury (2018) [66]	Meta-analysis	Eight studies were included in quantitative analysis (total *n* = 1644, mean age ranged from 39 to 57 years, % of females ranged from 76.6% to 100%)	- Moderate association between poor adherence to GFD and greater depressive symptoms (*r* = 0.398, 95% CI: 0.32–0.47) with marked heterogeneity in effects (*I*^2^ = 66.8%) - Exclusion of studies with high or unclear risk of bias did not alter the results	- Poorer QOL was correlated with a higher incidence of psychological and gastrointestinal symptoms, greater reliance on maladaptive coping strategies, and poorer GFD adherence
Feeding and eating disorders				
Passananti (2013) [67]	Cross-sectional	CD, *n* = 100 (mean age 29 years, 72% females) HC, *n* = 100 (mean age 30 years, 68% females)	- BES ≥ 17: 6% in CD vs. 0% controls (*p* = NS) - Women with CD had significantly higher scores in pulse thinness, social insecurity, perfectionism, inadequacy, ascetisim, and interpersonal diffidence compared to HC women of the Eating Disorder Inventory - EAT-26 ≥ 20: 16% in CD vs. 4% in HC (*p* = 0.01) - SRDS > 44: 39% in CD vs. 6% in controls (*p* <0.001) - SCL-90 pathological scores: 42% in CD vs. 6% in HC (*p* <0.0001)	- EAT-26 demonstrated association between indices of diet-related disorders in both CD and the female gender after controlling for anxiety and depression
Satherley (2016) [68]	Cross-sectional	CD, *n* = 157 (mean age 38 years, sex NR) IBD, *n* = 116 (mean age 36 years, sex NR) DM-type 2, *n* = 88 (mean age 47 years, sex NR) HC, *n* = 142 (mean age 33 years, sex NR)	- EAT-26 > 20: 15.7% in CD vs. 8.8% in DM and 3.8% in HC (*p* <0.05) - BES > 17: 19.4% in CD vs. 2.3% in controls (*p* <0.05) - Mean EAT-26 and BES scores: 11.1 in CD vs. 7.7 in controls (*p* <0.05) and 11.2 in CD vs. 3.9 in controls (*p* <0.05), respectively - Significant associations between EAT-26 and BES scores with DASS-21 scores were reported (*p* <0.008)	- Dietary-management and gastrointestinal symptoms were significantly associated with EAT scores in CD
Mårild (2017) [69]	Population-based cohort study	Group 1, *n* = 17,959 (median age 28 years); Matched controls, *n* = 89,379 Group 2, *n* = 7455 (median age 46 years); Matched controls, *n* = 36,940 Group 3, *n* = 2307 (median age 38 years); Matched controls, *n* = 11,499	- Risk of developing anorexia nervosa: Group 1: HR= 1.46 (95% CI: 1.08–1.98) Group 2: HR=2.12 (95% CI: 0.97–4.67) Group 3: HR=2.45 (95% CI: 1.10–5.45) - Adjustment for education level, socioeconomic status, and type 1 DM didn’t affect conclusions in all groups	- There was no significantly increased risk for subsequent anorexia nervosa among males with CD
Sleep-Wake disorders				
Mårild (2015) [70]	Population-based cohort study	CD, *n* = 2933 (median age 28 years, 61.2% females) Controls, *n* = 14,571 (median age 28 years, 61.3 females)	- Poor sleep in CD vs. controls: 12.5% vs. 9.8% (HR = 1.36, 95% CI: 1.30–1.41) - Individuals with CD had a similar increased risk irrespective of age at CD diagnosis, sex and type of hypnotic used	- Overall, poor sleep was more prevalent in females than in males. However, differences in risk estimates for poor sleep were small between females and males with CD - Adjustment for sleep apnea and restless leg syndrome did not influence the risk of poor sleep in CD
Substance-related and addictive disorders				
Roos (2006) [71]	Cross-sectional	CD, *n* = 51 (age 45–64 years, 59% females) Controls, *n* = 182 (age 45–64 years, 57% females)	- PGWB index scores: 103 (95% CI: 99–107) in CD vs. 103 (95% CI: 100–106) in controls (*p* = NS)	- Males with CD tended to score higher on the PGWB domains than the male controls - CD women scored somewhat lower in the PGWB domains than the female controls - CD men tended to score higher than the CD women in all six domains of the PGWB
Gili 2013 [72]	Cross-sectional	CD, *n* = 3327 (mean age 49 years and 70% females). Controls, *n* = 5,471,988) (mean age 58 years and 54% females).	- Prevalence of alcohol disorders: 4.9% in CD vs. 6.3% in controls (*p* = 0.0009)	- The presence of alcohol disorders in CD increased the length of stay, costs and had an excess of mortality
Neurocognitive Disorders				
Lebwohl (2016) [73]	Population-based cohort	CD, *n* = 8846 (mean age 64 years and 56% females). Control, *n* = 43,474 (mean age 64 years and 56% females).	- In a median follow-up period of 8.4 years: 4.3% of CD patients and 4.4% of controls had a diagnosis of dementia (HR 1.07; 95% CI 0.95–1.20) - A subgroup analysis showed an increased risk of vascular dementia (HR 1.28; 95% CI 1.00–1.64)	- A significant association between CD and dementia among the age group 60–69 was found, which was not present in the younger or older age groups - Increased risk of dementia was found in the first year following CD diagnosis
Various psychiatric conditions				
Fera (2003) [74]	Cross-sectional	CD, *n* = 100 (mean age 40 years, 75% females) DM, *n* = 100 (mean age 53 years, 74% females) HC, *n* = 100 (mean age 41 years, 68% females)	- CD, prevalence of OCD 28%, depressive disorder/dysthymia 19% - DM, prevalence of OCD 0%, depressive disorder/dysthymia 10% HC, anxiety and depression in 10% of subjects	- QOL was poorer in both CD and diabetic patients than in healthy controls and significantly correlated with anxiety
Sainsbury (2013) [75]	Study 1: cross-sectional	*n* = 390 (mean age 44 years, 82.8% females)	- Severe gastrointestinal symptoms at CD diagnosis were associated with: increased depression (*r* = 0.28, *p* <0.001), anxiety (*r* = 0.29, *p* <0.001), stress (*r* = 0.28, *p* <0.001), eating disorder (*r* = 0.15, *p* <0.01), and emotion-oriented coping (*r* = 0.17, *p* <0.01)	- Poorer QOL was significantly associated with a greater number and longer duration of CD symptoms prior to diagnosis - Higher number of symptoms was associated with poorer QOL - There were no gender differences in QOL, although females reported a greater number of symptoms - More severe gastrointestinal symptoms at diagnosis were also associated with increased psychological manifestations
	Study 2: cross-sectional	*n* = 189 (mean age 46.5 years, 87.3% females)	- Hierarchical regression analyses: Current psychological distress significantly contributed to poor QOL (accounting for 23.8% of the variance in QOL)	
Zylberberg (2017) [76]	Population-based cross-sectional	Diagnosed CD, *n* = 27 (age NR, 78% females) Undiagnosed CD, *n* = 79 (age NR, 58% females) PWAG; *n* = 213 (age NR, 55% females) Controls; *n* = 14,769 (demographic characteristics NR)	- Prevalence of depression: 8.2% of controls vs. 3.9% in CD (*p* = 0.18) and 2.9% in PWAGs (0.002) - Prevalence of sleep difficulty: 37.3% in CD, 34.1% in PWAGs vs. 27.4% in controls (*p* = NS) - Multivariate analysis adjusted for race/ethnicity, annual household income, number of healthcare visits: PWAGS, significantly lower odds of depression (OR = 0.25, 95% CI: 0.12–0.5, *p* = 0.0001) CD, OR = 0.30; 95% CI: 0.08–1.19, *p* = 0.09	- QOL: The presence of physical, mental, and emotional limitations was reported in 2.9% of controls vs. 13.8% diagnosed CD (*p* =0.004), 9.6% with undiagnosed CD (*p* = 0.02), and 5.1% in PWAGs (*p* = 0.18)

ADHD: Attention-Deficit Hyperactivity Disorder; ASD: Autistic Spectrum Disorders; BES: Binge Eating Scale; CD: celiac disease; CI: confidence interval; CPH: Chronic persistent hepatitis; DASS: Depression Anxiety Stress Scale; DM: Diabetes Mellitus; EAT: Eating Attitudes Test; EPDS: Edinburgh Postnatal Depression Scale; GFD: gluten-free diet; HC: healthy controls; HR: hazard ratio; IBD: Inflammatory Bowel Disease; IBS: Irritable Bowel Syndrome; MMPI: Minnesota Multiphasic Personality Inventory; M-SDS: Modified Zung Self-Rating Depression Scale; NCGS: non-celiac gluten sensitivity; NR: not reported; NS: not significant; OCD: Obsessive-Compulsive Disorder; OR: odd ratio; PGWB: Psychological General Well-being; PWAG: people who avoid gluten; QOL: quality of life; QOL: quality of life; RR: relative risk; SCL: Symptom Check List; SRDS: Zung Self-Rating Depression Scale; vs: versus.

## Supplementary Materials

The supplementary materials are available at: http://www.mdpi.com/2072-6643/10/7/875/s1. Table S1: Database specific search strategies.
