# Supplementary material for: Psychiatric Comorbidity in Children and Adults with Gluten-Related Disorders: A Narrative Review
Source: Nutrients. 2018 Jul 6;10(7):875. doi: 10.3390/nu10070875 (PMC6073457; doi:10.3390/nu10070875)
Supplement: Supplementary file 1 [file nutrients-10-00875-s001.zip › nutrients-320213-SI.pdf]

Psychiatric comorbidity in children and adults with gluten-related disorders: a narrative review

Supplementary Table 1. Database specific search strategies

Supplementary Table 1.1. Medline

**Mental disorders term**	MeSH headings	mental disorders
		**AND**
**Celiac disease or gluten related disorder term**	Keywords searched for in abstract and title	gluten OR non-celiac gluten sensitivity OR gluten intolerance OR celiac disease OR celiac sprue OR gluten enteropathy OR nontropical sprue OR wheat hypersensitivity OR gluten ataxia OR dermatitis herpetiformis OR wheat allergy OR wheat intolerance OR coeliac disease

Supplementary Table 1.2. EMBASE

**Mental disorders term**	Emtree subject headings	mental disease
		**AND**
**Celiac disease or gluten related disorder term**	Keywords searched for in abstract and title	gluten OR non-celiac gluten sensitivity OR gluten intolerance OR celiac disease OR celiac sprue OR gluten enteropathy OR nontropical sprue OR wheat hypersensitivity OR gluten ataxia OR dermatitis herpetiformis OR wheat allergy OR wheat intolerance OR coeliac disease

Supplementary Table 1.3. PsycINFO

**Mental disorders term**	APA Thesaurus	mental disorders
		**AND**
**Celiac disease or gluten related disorder term**	Keywords searched for in abstract and title	gluten OR non-celiac gluten sensitivity OR gluten intolerance OR celiac disease OR celiac sprue OR gluten enteropathy OR Nontropical sprue OR wheat hypersensitivity OR gluten ataxia OR dermatitis herpetiformis OR wheat allergy OR wheat intolerance OR coeliac disease
